# Association between insomnia phenotypes and subclinical myocardial injury: the Multi-Ethnic Study of Atherosclerosis

**DOI:** 10.1093/sleep/zsac318

**Published:** 2022-12-29

**Authors:** Fjola D Sigurdardottir, Suzanne M Bertisch, Michelle L Reid, Christopher R deFilippi, Joao A C Lima, Susan Redline, Torbjørn Omland

**Affiliations:** Department of Cardiology, Akershus University Hospital, Lørenskog, Norway; Institute of Clinical Medicine, University of Oslo, Oslo, Norway; Division of Sleep and Circadian Disorders, Brigham and Women’s Hospital, Boston, MA, USA; Division of Sleep Medicine, Harvard Medical School, Boston, MA, USA; Division of Sleep and Circadian Disorders, Brigham and Women’s Hospital, Boston, MA, USA; Inova Heart and Vascular Institute, Inova Fairfax Medical Campus, Falls Church, VA, USA; Division of Cardiology, Department of Medicine, Johns Hopkins Hospital and School of Medicine, Baltimore, MD, USA; Division of Sleep and Circadian Disorders, Brigham and Women’s Hospital, Boston, MA, USA; Division of Sleep Medicine, Harvard Medical School, Boston, MA, USA; Department of Cardiology, Akershus University Hospital, Lørenskog, Norway; Institute of Clinical Medicine, University of Oslo, Oslo, Norway

**Keywords:** insomnia, COMISA, subclinical myocardial injury, cardiac troponin

## Abstract

**Study Objectives:**

To assess whether the association between insomnia and subclinical myocardial injury, as measured by cardiac troponin T (cTnT), differs across insomnia phenotypes.

**Methods:**

We measured cTnT in 2188 participants in the Multi-Ethnic Study of Atherosclerosis study who had completed sleep questionnaires and undergone unattended polysomnography (PSG) and 7-day actigraphy. Insomnia symptoms were defined as reporting at least one of the following ≥5 nights/week over the past 4 weeks: trouble falling asleep, waking up several times a night, having trouble getting back to sleep after waking up too early, or taking sleeping pills to help falling asleep. Obstructive sleep apnea (OSA) was defined as an apnea-hypopnea index (AHI >15 events/h). Participants were classified into insomnia phenotypes, including comorbid insomnia and OSA (COMISA) and insomnia associated with actigraphy-estimated short sleep (<6 h) or sleep fragmentation.

**Results:**

The mean age was 68.8 (*SD* 9.2) years, 53.6% were male. In total, 47.8% met threshold levels for insomnia symptoms, and 43.1% had an AHI >15. In adjusted linear regression models COMISA (β 0.08 [standard error (*SE*) 0.03], *p* < .01) and insomnia with short sleep duration (β 0.07 [*SE* 0.03], *p* < .05) were each associated with higher cTnT compared to a reference group with no insomnia. Insomnia with fragmented sleep (β 0.03 [*SE* 0.02]) was not associated with higher cTnT (*p* > .05) in adjusted analyses. OSA was associated with higher cTnT (β 0.09 [SE 0.03], *p* < .01) in adjusted models.

**Conclusions:**

COMISA and insomnia with short sleep duration, but not insomnia symptoms alone or fragmented sleep, were associated with increased circulating cTnT in older adults.

## Graphical Abstract

**Graphical Abstract:**
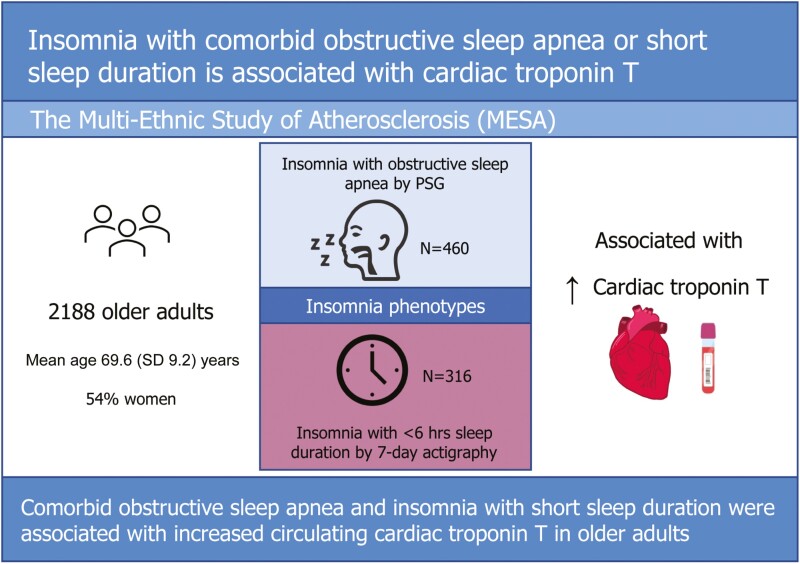
Picture vectors purchased on 123rf.com

**Statement of Significance**

Insomnia symptoms have been linked to an increased risk of cardiovascular disease (CVD), heart failure (HF), and mortality in some studies, but results are inconsistent. A potential explanation for these inconsistent findings is that insomnia symptoms often co-occur with other sleep disorders that are independently associated with CVD, but few studies on insomnia have objective data on other sleep characteristics.

This is the first study to assess the association of three different insomnia phenotypes with subclinical myocardial injury as measured by cardiac troponin T (cTnT): insomnia with comorbid obstructive sleep apnea (OSA), also known as COMISA; insomnia with objectively short sleep duration; and insomnia with fragmented sleep. We also assessed the association between OSA without insomnia with cTnT.

## Introduction

Insomnia symptoms are the most common sleep complaint in the general population. The prevalence ranges from 7.9% to 33% and may be even more prevalent in socioeconomically disadvantaged populations [1]. Self-reported insomnia symptoms are associated with a higher risk of cardiovascular disease (CVD) in epidemiological studies [2–4]. However, it is unclear what specific role insomnia symptoms play in the pathophysiology of CVD and whether the observed epidemiological associations might be attributable to other co-occurring sleep characteristics, such as short sleep duration or obstructive sleep apnea (OSA) [3]. Between 29% and 67% of individuals with insomnia symptoms have comorbid OSA when tested with polysomnography (PSG) [5]. Few epidemiological studies evaluating the association between insomnia and CVD risk have collected objective data about OSA with overnight PSG and have instead relied on body mass index (BMI) or self-reported snoring as a proxy. Thus, the effect of undiagnosed OSA on this association remains unclear.

Cardiac troponin T (cTnT) is a biomarker of subclinical myocardial injury associated with fibrosis on cardiovascular magnetic resonance imaging and increased risk of heart failure (HF) and CVD death [6]. We previously reported that cardiac troponin I (cTnI), a related but physiologically distinct circulating biomarker of subclinical myocardial injury, is increased in individuals with insomnia symptoms in an epidemiological cohort without objective data on OSA in crude, but not adjusted analysis [7]. However, the association between insomnia symptoms and CVD may differ across different insomnia phenotypes. For example, in data from the Sleep Heart Health Study (SHHS), insomnia with short sleep duration on single-night PSG was found to be associated with a 29% higher risk of CVD, but neither insomnia nor short sleep duration alone was significantly associated with higher risk [3].

Although the pathophysiological mechanisms whereby insomnia contributes to CVD risk remains to be delineated, direct recordings of sympathetic neural activity suggest that sympathetic neural hyper-reactivity is one potential mechanism through which insomnia symptoms, often characterized to be a result of a state of hyperarousal, might contribute to CVD risk [8]. Increased sympathetic activity is also seen in HF patients who may have chronically elevated cTnT. Chronically increased sympathetic nervous system activation is detrimental, contributing to cardiac dysfunction, myocardial injury, higher mortality, and morbidity in HF, while inhibition of beta-adrenoceptor-mediated pathways in HF reduces mortality [9, 10].

Accordingly, we hypothesized that the association between insomnia and subclinical myocardial injury measured by cTnT may differ across insomnia phenotypes. To test this hypothesis, we assessed the association between insomnia symptoms and subclinical myocardial injury in a heterogeneous population while considering objective data on associations between different insomnia phenotypes, including short sleep duration and OSA, and cTnT.

## Methods

The Multi-Ethnic Study of Atherosclerosis (MESA) is a multicenter, community-based cohort study of 6814 participants without a known history of CVD at the baseline examination. The study was conducted at six centers in the United States and was designed to oversample individuals from diverse racial/ethnic groups (38% White, 28% Black, 22% Hispanic, and 12% Chinese-American). MESA collected information on sociodemographics, health habits, and medical history using questionnaires and standardized interviews at several exams since the beginning of the study in 2000–2002 with the baseline exam (Exam 1) and follow-up exams about every subsequent 2 to 6 years. The current study represents the analysis of participants at Exam 5 (2010–2012) who also participated in the MESA Sleep Ancillary study (MESA-Sleep, 2010–2013). The MESA-Sleep study collected information on sleep using at-home PSG, 7-day wrist actigraphy, and sleep questionnaires. Participants who reported the use of regular use of oral devices, nocturnal oxygen, or use of positive airway pressure were not included in the MESA-Sleep study. In total, 2261 (59.7%) of the 3789 participants that were approached and did not fulfill any exclusion criteria participated in MESA Sleep. Successful PSG data were collected in 2057 participants, actigraphy data in 2156 participants, and sleep questionnaires in 2240 participants; 2188 participants (with complete insomnia data) had cTnT measured during Exam 5 [11]. cTnT was measured with a high-sensitivity assay in ethylenediaminetetraacetic acid plasma, collected with a morning blood sample at Exam 5, using the Cobas e602 (Roche Diagnostics, Rotkreuz, Switzerland) at the Inova Biocore laboratory, Fall Church Virginia, United States. The limit of detection (LOD) of the assay was 3.0 ng/L. Coefficient of variation at different concentrations for the MESA cohort was 3.6% at 28 ng/L and 2% at 254 ng/L [6]. Estimated glomerular filtration rate (eGFR) was calculated using the Chronic Kidney Disease Epidemiology Collaboration (CKD-EPI) equation [6, 12]. All participants provided written informed consent. The dataset used for these analyses can be accessed by reasonable request to MESA Publication and Presentations Committee (https://www.mesa-nhlbi.org).

PSG was performed at home using a portable 15-channel monitor (Compumedics Somte System; Compumedics Ltd., Abbotsville, Australia) and scored according to the American Academy of Sleep Medicine Manual guidelines [11, 13]. The recording setup included electroencephalography, a chin electromyography, bipolar electrocardiography, thoracic and abdominal respiratory inductance plethysmography, airflow measured by thermocouple and nasal pressure cannula, and finger pulse oximetry in addition to bilateral electrooculograms and piezo sensors for limb movements. The apnea-hypopnea index (AHI) was calculated as the average number of apneas and hypopneas associated with a 3% desaturation per hour of sleep. Actigraphy was performed with an Actiwatch Spectrum wrist actigraphy (Philips Respironics, Murrysville, PA) worn for 7 consecutive days, while completing a sleep diary over the same period and processed using methods previously described [14, 15]. Overnight sleep duration of less than 6 h on actigraphy was defined as short sleep duration. Fragmented sleep was defined based on previous studies as an average Sleep Fragmentation Index of >20% derived from the actigraphy-based algorithm that combines the time spent with movement and the percentage of the number of immobile phases that were shorter than 1 min long [16, 17]. A detailed description of the PSG and actigraphy scoring protocol can be a found at sleepdata.org/datasets/mesa. Participants with PSG data had at least 7 h of scorable PSG recording. One hundred ninety-one participants had missing PSG recordings due to inability to collect minimal data and were therefore not included in the analyses that required PSG-recorded sleep duration or AHI. There was a median lag of 1 year between the blood sample draw at Exam 5 and the PSG measurements.

Insomnia was defined as a self-report of more than 5 nights/week over the past 4 weeks of at least one of the following symptoms included in the validated Women’s Health Insomnia Rating Scale [18]: “Did you have trouble falling asleep?,” “Did you wake up several times a night?,” “Did you have trouble getting back to sleep after you woke up too early?,” and the question “Did you take sleeping pills to help you sleep?,” providing a definition of insomnia symptoms consistent with prior epidemiologic studies [3, 19].

Baseline data are reported as absolute numbers (proportion) or median (25th percentile; Q1 and 75th percentile; Q3). Continuous variables were analyzed using the Mann–Whitney U-test, and categorical variables with the Pearson Chi-Square test and the Fisher exact test, as appropriate. Comparisons of groups were performed by testing for differences between insomnia and no insomnia overall in addition to gender-stratified analyses (Tables 1 and 2). Due to a skewed distribution, levels of cTnT were transformed by the natural logarithm prior to analyses. Linear and logistic regression analyses were used to assess the association between insomnia and logarithmically transformed cTnT, adjusting for relevant confounders such as gender, age, race/ethnicity, smoking, BMI, eGFR, and the AHI. Similar approaches were used to test the associations with PSG/actigraphy measures of several phenotypes of insomnia, specifically: insomnia with short sleep duration (less than 6 h per night on the actigraphy), insomnia with fragmented sleep (average fragmentation index >20% on actigraphy), and comorbid insomnia and OSA (COMISA). COMISA was defined as having both symptoms of insomnia and OSA as defined by an AHI >15 measured by PSG. All regression models were adjusted for gender (not adjusted for when stratified by gender), age, race/ethnicity, smoking, BMI, eGFR, and AHI (except for when looking at the association of COMISA phenotype). When looking at the association between OSA and cTnT, the regression models were additionally adjusted for insomnia in Model 4. As several traditional CVD risk factors such as hypertension, dyslipidemia, and diabetes are variables that could possibly be on the causal pathway between the association of insomnia phenotypes and CVD, we have not adjusted for these in our models [20–22]. In addition, analyses were conducted for individual phenotypes, stratified by gender (Supplementary Tables 1 and 2). Secondary analyses were performed using different definitions of insomnia phenotypes, including using PSG-defined short sleep duration (<6 h), insomnia with PSG-defined elevated wake after sleep onset (WASO) over the mean (>94 min), and insomnia with PSG-defined elevated arousal index was defined as the highest arousal index quartile (>28.1); Supplementary Table 3. Statistical significance was assumed at *p* < .05. SAS 9.4 (SAS Institute, Inc., Cary, NC) was used for statistical analysis. All participants provided written informed consent.

**Table 1.** Baseline characteristics of participants, overall and stratified by gender

	Overall		*P*	Males		*P*	Females		*P*
	Insomnia (*N* = 1046)	No insomnia (*N* = 1142)		Insomnia (*N* = 503)	No insomnia (*N* = 512)		Insomnia (*N* = 543)	No insomnia (*N* = 630)	
Age (years)	69.9 ± 9.4	67.4 ± 8.8	<.0001	70.6 ± 9.4	66.9 ± 8.7	<.001	69.3 ± 9.4	67.8 ± 9.0	.0056
Race/ethnicity			<.0001			<.001			<.001
White	429 (41.0%)	389 (34.1%)		204 (40.6%)	175 (34.2%)		225 (41.4%)	214 (34.0%)	
Chinese	77 (7.4%)	180 (15.8%)		45 (9.0%)	83 (16.2%)		32 (5.9%)	97 (15.4%)	
African American	277 (26.5%)	320 (28.0%)		115 (22.9%)	150 (29.3%)		162 (29.8%)	170 (27.0%)	
Hispanic	263 (25.1%)	253 (22.2%)		139 (27.6%)	104 (20.3%)		124 (22.8%)	149 (23.7%)	
Education			.9855			.3316			.4207
High school or lower	154 (14.8%)	167 (14.7%)		75 (14.9%)	61 (11.9%)		79 (14.6%)	106 (16.9%)	
Some college/technical school	424 (40.6%)	460 (40.4%)		178 (35.5%)	195 (38.2%)		246 (45.4%)	265 (42.1%)	
College graduate	466 (44.6%)	513 (45.0%)		249 (49.6%)	255 (49.9%)		217 (40.0%)	258 (41.0%)	
BMI (kg/m^2^)	29.2 ± 5.8	28.3 ± 5.4	.0005	28.6 ± 4.9	28.1 ± 4.5	.0756	29.7 ± 6.5	28.5 ± 6.0	.0017
Current smoker (%)	69 (6.7%)	76 (6.7%)	.0713	38 (7.6%)	34 (6.7%)	.3524	31 (5.8%)	42 (6.8%)	.1616
Physical activity (MET-min/week)	5037.3 ± 5493.8	5676.3 ± 7022.7	.0178	5477.2 ± 6190.7	6558.2 ± 8632.4	.0222	4630.1 ± 4728.1	4954.4 ± 5255.1	.2682
Depressive symptoms (CES-D > 16)	160 (16.8%)	122 (12.5%)	.0079	70 (15.5%)	46 (10.5%)	.0263	90 (18.0%)	76 (14.2%)	.0962
Diastolic blood pressure (mmHg)	67.8 ± 9.8	68.5 ± 10.2	.0844	70.0 ± 9.7	71.6 ± 10.0	.0105	65.7 ± 9.5	66.0 ± 9.7	.5748
Systolic blood pressure (mmHg)	123.4 ± 19.9	122.3 ± 20.9	.2003	122.1 ± 19.6	121.8 ± 19.5	.8060	124.6 ± 20.2	122.6 ± 22.1	.1178
HDL cholesterol (mg/dL)	56.2 ± 16.9	54.7 ± 15.7	.0404	49.5 ± 13.6	49.1 ± 13.1	.6374	62.3 ± 17.3	59.3 ± 16.2	.0020
Total cholesterol (mg/dL)	182.9 ± 36.9	184.0 ± 37.1	.4912	171.4 ± 35.1	172.2 ± 35.7	.7039	193.6 ± 35.4	193.6 ± 35.5	.9925
Current diabetes (%)	223 (21.3%)	210 (18.4%)	.1640	113 (22.5%)	102 (19.9%)	.4193	110 (20.3%)	108 (17.1%)	.3836
Current cardiovascular disease (%)	123 (11.8%)	110 (9.6%)	.1087	79 (15.7%)	62 (12.1%)	.0976	44 (8.1%)	48 (7.6%)	.7645
Current Atrial fibrillation on 12 lead ECG (%)	14 (1.4%)	8 (0.7%)	.1382	9 (1.8%)	4 (0.8%)	.1733	5 (0.9%)	4 (0.6%)	.7408
PSG based measurements									
Number of PLM per hour of sleep	16.4 ± 27.6	12.3 ± 22.5	.0004	20.9 ± 34.0	14.0 ± 24.8	.0004	12.2 ± 19.0	10.9 ± 20.3	.3139
Time in bed (min)	482.7 ± 84.7	472.4 ± 80.1	.0054	473.5 ± 87.6	459.9 ± 78.9	.0134	491.1 ± 81.0	482.6 ± 79.8	.0849
Total sleep time (min)	357.7 ± 84.2	362.1 ± 81.1	.2417	336.8 ± 83.8	345.3 ± 74.4	.1047	377.1 ± 79.9	375.9 ± 83.7	.8123
Short sleep duration (<6 h)	446 (47.1%)	459 (43.7%)	.1295	263 (57.8%)	250 (52.7%)	.1211	183 (37.2%)	209 (36.3%)	.7583
Total recording time (hr)	10.6 ± 1.5	10.5 ± 1.5	.0563	10.6 ± 1.6	10.4 ± 1.6	.1991	10.7 ± 1.4	10.6 ± 1.4	.1313
Apnea-hypopnea index (PSG-based AHI), events/h.	20.4 ± 19.3	19.2 ± 18.1	.1509	25.4 ± 20.5	23.7 ± 19.9	.1935	15.8 ± 16.8	15.5 ± 15.6	.7835
AHI > 15 events/h	460 (48.6%)	482 (45.9%)	.2327	273 (60.0%)	269 (56.8%)	.3153	187 (38.0%)	213 (37.0%)	.7291
Arousal Index arousal/h	23.3 ± 12.7	21.5 ± 11.5	.0011	26.6 ± 14.0	24.4 ± 12.5	.0128	20.2 ± 10.6	19.1 ± 10.0	.0727
Sleep efficiency (%)	74.5 ± 13.7	76.9 ± 13.1	<.0001	71.6 ± 14.6	75.5 ± 12.8	<.001	77.1 ± 12.4	78.1 ± 13.2	.2287
WASO	103.5 ± 68.4	86.6 ± 61.2	<.0001	115.8 ± 72.5	94.5 ± 61.7	<.001	92.2 ± 62.3	80.2 ± 60.0	.0014
Actigraphy based measurements									
Fragmented sleep (>20%)	470 (44.9%)	447 (39.1%)	.0061	275 (54.7%)	259 (50.6%)	.1928	195 (35.9%)	188 (29.8%)	.0271
Average sleep duration (min)	387.7 ± 86.4	390.2 ± 76.2	.4884	374.3 ± 93.0	374.3 ± 75.9	.9903	400.0 ± 78.0	403.0 ± 74.1	.5033
Short sleep duration (<6 h)	316 (31.5%)	335 (30.9%)	.7896	175 (36.3%)	183 (37.7%)	.6464	141 (27.0%)	152 (25.4%)	.5450
Sleep efficiency (%)	89.5 ± 3.8	90.1 ± 3.6	.0009	88.9 ± 4.3	89.5 ± 3.7	.0161	90.1 ± 3.2	90.5 ± 3.4	.0442
WASO	38.6 ± 17.0	36.7 ± 16.0	.0008	39.9 ± 18.9	37.1 ± 15.9	.0118	37.4 ± 14.9	35.4 ± 16.1	.0353

BMI; body mass index; MET; metabolic equivalent of the task; CES-D; Center for Epidemiological Studies Depression Scale; PSG, polysomnography; AHI, apnea-hypopnea index; HDL, high-density lipoprotein. Insomnia, is defined as more than 5 nights/week over the past 4 weeks of trouble falling asleep, waking up several times a night, trouble getting back to sleep, or taking sleep medication. WASO; Wake after Sleep onset.

**Table 2.** Impact of different cTnT cutoffs on associations with insomnia symptoms

	Overall		*P*	Males		*P*	Females		*P*
	Insomnia (*N* = 1046)	No Insomnia (*N* = 1142)		Insomnia (*N* = 503)	No insomnia (*N* = 512)		Insomnia (*N* = 543)	No insomnia (*N* = 630)	
cTnT median (IQR) ng/L	8.9 (6.4, 13.3)	8.0 (5.8, 11.5)	.0646	10.9 (8.0, 16.4)	9.5 (7.2, 14.6)	.0170	7.4 (5.4, 10.7)	6.8 (5.1, 9.5)	.7947
cTnT ≥ LOD (3 ng/L)	1039 (99.3%)	1126 (98.6%)	.0936	502 (99.8%)	510 (99.6%)	1.0	537 (98.9%)	616 (97.8%)	.1405
cTnT > 99th percentile ≥11.8 ng/L females; ≥ 18.5 ng/L males	204 (19.5%)	161 (14.1%)	.0007	102 (20.3%)	71 (13.9%)	.0066	102 (18.8%)	90 (14.3%)	.0378
cTnT > 99th percentile (Universal) ≥ 16.9 ng/L	159 (15.2%)	132 (11.6%)	.0122	118 (23.5%)	91 (17.8%)	.0251	41 (7.6%)	41 (6.5%)	.4850

## Results

Of the analytical sample, 1046 (47.8) reported insomnia symptoms. Of those who met the threshold for insomnia symptoms, 543 (52%) were women. Participants with insomnia were older than those without insomnia, had higher BMI, were less physically active, and had a higher prevalence of depressive symptoms, self-reported diabetes, and CVD (Table 1). By PSG, individuals with insomnia also had higher arousal index, more periodic limb movements (PLM), lower sleep efficiency, and more WASO but no differences in sleep duration (Table 1). By actigraphy, individuals with insomnia had more fragmented sleep, lower sleep efficiency, and higher WASO but no differences in sleep duration (Table 1). The prevalence of insomnia symptoms did not differ for those with or without OSA. Similar patterns were seen for men and women. The average total PSG recording time was 10.5 (*SD* 1.5) hrs.

Median cTnT was 8.9 (interquartile range [IQR]: 6.4–13.3) ng/L in participants with insomnia symptoms as compared to 8.0 (IQR: 5.8–11.5) ng/L in those without insomnia symptoms (Table 2). There was a high prevalence of cTnT values over the LOD in the total sample. The vast majority (98.9%) of participants had cTnT concentrations greater or equal to 3 ng/L. Compared to those without insomnia, both men and women with insomnia were more likely to have cTnT over the sex-specific 99th percentile (18.5 ng/L for men and 11.8 ng/L for women) (19.5% vs 14.1%) (Table 2). There was a significant association between the presence of insomnia symptoms and cTnT in unadjusted models, but after adjusting for sex, age and race/ethnicity, and smoking, no association was observed (Table 3).

**Table 3.** Linear association between insomnia symptoms and logarithmically transformed cTnT among participants with measurable cTnT

	Overall	Females	Males
	Beta (*SE*)	Beta (*SE*)	Beta (*SE*)
Model 1	0.12 (0.02)^**^	0.09 (0.03)^**^	0.13 (0.03)^**^
Model 2	0.03 (0.02)	0.04 (0.03)	0.02 (0.03)
Model 3	0.01 (0.02)	0.02 (0.03)	−0.002 (0.03)
Model 4	0.01 (0.02)	0.03 (0.03)	−0.009 (0.03)

Model 1: Unadjusted,

Model 2: Sex, age, race/ethnicity, smoking (no adjustments for sex in gender stratified analysis).

Model 3: Model 2 + BMI.

Model 4: Model 3 + eGFR, AHI.

^**^ *P* < .01.

We further evaluated the association between different insomnia phenotypes and continuous log-transformed cTnT values. In unadjusted analyses, higher circulating cTnT concentration was associated with insomnia symptoms without regard to other sleep characteristics β 0.12 (standard error [*SE*] 0.03, *p* < .01); insomnia symptoms with actigraphy-defined short sleep duration β 0.24 (*SE* 0.04, *p* < .01); and insomnia with actigraphy-defined fragmented sleep β 0.27 (*SE* 0.03, *p* < .01). After adjustments for sex, age, race/ethnicity, smoking, and BMI the associations with insomnia symptoms alone and insomnia with fragmented sleep were attenuated, and only the associations with insomnia COMISA (β 0.07 [*SE*] 0.03, *p* < .01) remained significant (Table 4). As a comparator, OSA (without regard to the presence of insomnia) was also significantly associated with increased circulating cTnT concentrations in fully adjusted models (β 0.05 [*SE*] 0.02, *p* < .05), even after adjusting for insomnia. (Table 4) Moreover, in fully adjusted models, including AHI, actigraphy-defined short sleep duration was associated with cTnT (β 0.07 [*SE*] 0.03, *p* < .01) (Table 4). Analysis stratified by sex did not reveal substantive differences compared to analyses conducted in the overall sample. While under-powered, sex-stratified analyses showed almost identical estimates for associations between COMISA and cTnT in men and women. The associations between COMISA and cTnT were significant in both men and women in all models except after adjusting for BMI (Supplementary Tables 1 and 2). The association between insomnia with short sleep duration and cTnT was only significant in men after adjusting for age, race/ethnicity, and smoking (Supplementary Tables 1 and 2).

**Table 4.** Association between insomnia symptoms and circulating cTnT in different insomnia phenotypes; insomnia symptoms, insomnia symptoms with actigraphy-defined short sleep duration (<6 h), insomnia with actigraphy-defined fragmented sleep, and COMISA with no symptoms of insomnia as a reference

	Model 1	Model 2	Model 3	Model 4
	β (*SE*)	β (*SE*)	β (*SE*)	β (*SE*)
No insomnia (*N* = 1142)	Ref	Ref	Ref	Ref
Insomnia (*N* = 1046)	0.12 (0.03)^**^	0.03 (0.02)	0.01 (0.02)	0.02 (0.02)
Model 1: Unadjusted Model 2: Sex, age, race/ethnicity, smoking Model 3: Model 2 + BMI Model 4: Model 3 + eGFR, AHI				
No insomnia with sleep duration (≥ 6 h) (*N* = 849)	Ref	Ref	Ref	Ref
No insomnia with short sleep duration (<6 h) (*N* = 335)	0.06 (0.04)	0.04 (0.03)	0.03 (0.03)	0.05 (0.03)
Insomnia with sleep duration (≥6 h) (*N* = 688)	0.09 (0.03)^**^	0.01 (0.02)	0.005 (0.02)	0.01 (0.02)
Insomnia with short sleep duration (<6 h) (*N* = 316)	0.24 (0.04)^**^	0.08 (0.03)^**^	0.05 (0.03)	0.07 (0.03)^*^
Model 1: Unadjusted Model 2: Sex, age, race/ethnicity, smoking Model 3: Model 2 + BMI Model 4: Model 3 + eGFR, AHI				
No insomnia with non-fragmented sleep (≤ 20%) (*N* = 695)	Ref	Ref	Ref	Ref
No insomnia with fragmented sleep (>20 %) (*N* = 447)	0.18 (0.03)^**^	0.06 (0.03)^*^	0.04 (0.03)	0.03 (0.03)
Insomnia with non-fragmented sleep (≤20%) (*N* = 576)	0.13 (0.03)^**^	0.04 (0.03)	0.02 (0.03)	0.02 (0.03)
Insomnia with fragmented sleep (>20%) (*N* = 470)	0.27 (0.03)^**^	0.06 (0.03)^*^	0.04 (0.03)	0.05 (0.03)
Model 1: Unadjusted Model 2: Sex, age, race/ethnicity, smoking Model 3: Model 2 + BMI Model 4: Model 3 + eGFR, AHI				
No insomnia with no-OSA (AHI ≤ 15) (*N* = 568)	Ref	Ref	Ref	Ref
No insomnia with OSA (AHI > 15) (*N* = 482)	0.22 (0.03)^**^	0.11 (0.03)^**^	0.05 (0.03)	—
Insomnia with no-OSA (AHI ≤ 15) (*N* = 487)	0.11 (0.03)^**^	0.02 (0.03)	0.007 (0.03)	—
Insomnia with OSA (AHI > 15) (*N* = 460)	0.32 (0.03)^**^	0.13 (0.03)^**^	0.07 (0.03)^*^	—
Model 1: Unadjusted Model 2: Sex, age, race/ethnicity, smoking Model 3: Model 2 + BMI ^*^N = 191 individuals with no AHI value				
No-OSA (AHI ≤ 15) (*N* = 1055)	Ref	Ref	Ref	Ref
OSA (AHI > 15) (*N* = 942)	0.22 (0.02)^**^	0.11 (0.02)^**^	0.05 (0.02)^*^	0.05 (0.02)^*^
Model 1: Unadjusted Model 2: Sex, age, race/ethnicity, smoking Model 3: Model 2 + BMI Model 4: Model 3 + insomnia ^*^N = 191 individuals with no AHI value				

^**^ *P* < .01,

^*^ *P* < .05.

In a secondary analysis, we explored insomnia phenotypes using PSG-defined attributes: short sleep duration (<6 h), elevated arousal index (>28.1), or high WASO (WASO > 94 min). No insomnia phenotype defined using these PSG metrics was associated with circulating cTnT (Supplementary Table 3). In a sensitivity analysis, we saw no association between short sleep by actigraphy and cTnT in unadjusted β 0.07 (*SE* [0.04], *p* > .05) or adjusted models (β 0.04 [*SE* 0.03], *p* > .05).

## Discussion

The new and important information derived from the current multi-ethnic community-based study is that cTnT concentrations differ across phenotypes of insomnia symptoms. Specifically, of four insomnia subtypes, we observed that only COMISA and insomnia with actigraphy-defined short sleep duration were significantly associated with cTnT in adjusted models. While there were significant associations between insomnia and insomnia with fragmented sleep with increased cTnT levels in crude analyses, these associations were no longer apparent after considering confounding with age, sex, race/ethnicity, and BMI. In summary, among an older community-based cohort with a high prevalence of both OSA and insomnia symptoms, only insomnia with comorbid OSA (COMISA) or insomnia with short sleep duration on actigraphy appeared to associate with subclinical myocardial injury as expressed by cTnT, suggesting that these phenotypes may be of special relevance to sleep-related susceptibility to CVD. However, we also observed an association between OSA and cTnT, and thus OSA may be a driving component of the association of COMISA with subclinical myocardial injury in older individuals from general community settings. In contrast, we saw no association with short sleep duration defined by actigraphy and cTnT, suggesting that for the insomnia-short sleep phenotype, it is the combination of perceived sleep problems (insomnia symptoms) and short sleep duration that together describe a clinically relevant phenotype.

The unique strength of this study is the objective and detailed phenotyping with actigraphy and PSG data that enabled us to define two common phenotypes: COMISA and insomnia with short sleep duration. These analyses showed that both phenotypes (COMISA and actigraphy-based short sleep and insomnia) were associated with circulating cTnT concentrations, while neither insomnia alone nor insomnia associated with any measure of sleep fragmentation (from actigraphy or PSG) was associated with this outcome. The null association with insomnia alone is consistent with the results of a previous population-based study where we also found no association between insomnia symptoms and circulating cTnI concentrations in adjusted models [7]. However, we did not have PSG or actigraphy data to address associations with insomnia subtypes that recently have been linked to all-cause mortality and increased risk of CVD in fully adjusted models [23, 24].

Our consistent finding that insomnia in general is not associated with increased circulating cTn, in addition to our novel finding that the COMISA and insomnia with short sleep duration phenotypes are associated with increased cTnT, underscores the importance of comprehensively assessing multiple aspects of sleep in studies of insomnia and CVD to distinguish associations that may be driven by co-occurring sleep disturbances or disease sub-phenotypes. Specifically, collecting only symptom-based measures does not provide the information needed to fully describe insomnia phenotypes such as COMISA, insomnia with short sleep duration, or other comorbid sleep conditions that may drive or confound associations with CVD. This is of particular concern in older persons because of the high prevalence of OSA, the limited ability of questionnaires to differentiate between insomnia symptoms and OSA [25], the established links between OSA and the risk of CVD [25–27], and misclassification of sleep duration using questionnaire alone [11]. Failure to account for OSA or sleep duration can lead to misspecification of the relationship between insomnia and CVD risk. Second, this study also highlights the importance of investigating various insomnia phenotypes. Insomnia symptoms are highly prevalent in the population. Identifying which phenotypes of insomnia might be more malignant than others may help target individuals at higher risk for further study or intervention. The associations we observed for OSA or COMISA with cTnT are consistent with prior literature that reported that cTnT and cTnI are increased in OSA and are associated with OSA severity [28–30]. Our findings of an association between COMISA and cTnT are consistent with a recent analysis of data from the SHHS that showed that COMISA was associated with higher CVD risk than insomnia alone [24]. Our data point to a biological pathway—subclinical myocardial injury—that may explain the findings from the SHHS.

A second phenotype, insomnia with objective short sleep duration, is also of interest as a potentially more malignant phenotype than insomnia alone, supported by a Mendelian randomization study from the UK biobank that provided evidence of a causal link between short sleep to hypertension, coronary artery disease, myocardial infarction, and chronic ischemic heart disease [3, 31]. In the Penn State cohort, insomnia with objective short sleep duration defined using PSG was associated with increased mortality in men with an odds ratio of 4.00 (95% CI = 1.14 to 13.99) after adjusting for potential confounders, but not in women [32]. Additionally, individuals with chronic insomnia with PSG-defined short sleep duration of 5–6 h had an increased risk of hypertension with an odds ratio of 3.53 (95% CI = 1.57 to 7.91) in the same cohort [33]. In the SHHS, however, symptoms of insomnia occurring with PSG-measured short sleep duration were associated with a 29% higher risk of CVD compared with individuals without insomnia or poor sleep and who slept at least 6 h [3]. We, therefore, hypothesized that insomnia with sleep duration <6 h would be associated with increased subclinical myocardial injury. However, we only saw an increase in subclinical myocardial injury in the insomnia phenotype with short sleep duration assessed by actigraphy in models adjusted for sex, age, race/ethnicity, smoking, BMI, eGFR, and AHI but not by PSG. Our data identified a higher prevalence of short sleep when single-night PSG was used compared to when defining sleep duration using 7-day average data from actigraphy. Therefore, in an older sample, actigraphy collected over several nights may capture habitual sleep patterns better than a single-night PSG. Compared to the SHHS and Penn State Cohort, the participants in the current study were significantly older, had a higher BMI, and had a higher prevalence of OSA, which may have influenced our results. It is also of interest that our participants with and without insomnia did not differ by total sleep duration using either PSG or actigraphy, but those with insomnia did have longer times in bed and lower sleep efficiency—a well-described sleep pattern with insomnia. We postulated that a third phenotype, insomnia, and objective sleep fragmentation, may have conferred higher risk of elevated cTnT than insomnia alone, but did not find evidence to support this hypothesis. Moreover, in the secondary analysis (Supplementary Table 3), we found no association between insomnia and PSG-defined high arousal index or insomnia with PSG-defined high WASO and cTnT. However, it is possible that more granular measures of sleep architecture may have identified new associations. Future studies prospectively examining associations and using quantitative measures of sleep macro- and micro-architecture may be useful. As an example, shorter rapid eye movement sleep has been associated with higher inflammation and increased mortality [34, 35], although associations of this feature with insomnia have not been examined.

cTn are a sensitive and specific marker of subclinical myocardial injury and are associated with incident HF, coronary heart disease, and mortality in the general population [6, 36–38]. However, it is important to recognize that there are multiple pathways that may link sleep disturbances to CVD. Inflammation is one critical pathway in early atherosclerosis and its progression to the clinical symptoms of significant atherosclerotic disease [30]. Insomnia symptoms have been reported to be significantly associated with higher levels of CRP in a cohort of Swedish women [34]. In another study from the MESA cohort, Bertisch *et al*. found that insomnia symptoms among women were significantly associated with an 18% higher prevalence of coronary calcification (CAC) compared to women without insomnia and this association did not appear to be mediated by OSA [19]. CAC is a specific marker of coronary atherosclerosis and is commonly used to assess the extent of subclinical atherosclerosis [19]. Based on these studies and our current findings, inflammation and subclinical atherosclerosis might be potentially important mechanisms driving the association between insomnia symptoms and CVD risk.

Several studies have found sex differences in the association of insomnia and insomnia phenotypes and future CVD risk [39–41]. We therefore also performed a gender-stratified sub-analysis for the association between insomnia phenotypes and cTnT. Our results showed similar association between the association of COMISA and OSA in men and women despite lower prevalence of OSA in women. However, there was not a significant association between COMISA and cTnT in a fully adjusted model that included adjustment for BMI. Although higher BMI is associated with OSA, insomnia, and higher cTnT values, and was adjusted for in our models, BMI might be part of the causal pathway between the association of COMISA and cTnT. BMI potentially being on the intermediating pathway in addition to underpowered analysis may explain the findings in our gender-stratified analysis where COMISA in women and men separately did not remain statistically significant after adjusting for BMI.

The study has several strengths. In addition to having both information on subclinical myocardial injury, self-reported information on insomnia symptoms, and objective measurements of insomnia phenotypes, our study is also the first study to investigate the association of insomnia symptoms using a sensitive assay of cTnT in a large multiracial/ethnic community cohort. Our study also has several limitations that warrant mentioning. First, we do not have data on the chronicity of insomnia symptoms or diagnostic interviews and our definitions do not fully reflect the current clinical diagnostic criteria for insomnia disorder which could have reduced the specificity of insomnia classification. It is also possible that patients presenting for clinical evaluation for insomnia may have more severe disease and may be at greater CVD risk than the participants in this community sample. Our sample was older, and our findings should not be generalized to younger individuals. Another important limitation is that due to the complexity of the data sampling, there was a lag between the blood sample collection and the PSGs that could have influenced our results. However, it has previously been shown that AHI change is limited over time and so are chronic cTnT levels [42, 43]. Additionally, this study cannot state causality as our findings only represent cross-sectional associations; however, we do not know of a mechanism by which subclinical myocardial injury (in the absence of clinical disease) would lead to insomnia.

## Conclusion

In this multi-ethnic cohort, we found that COMISA and insomnia with short sleep duration (<6 h) were associated with higher circulating levels of cTnT, a marker of subclinical myocardial injury. These findings suggest that OSA and short sleep duration may play a significant role in the pathophysiology of subclinical myocardial injury in individuals with insomnia and should be considered as common co-morbid sleep problems in older individuals with insomnia symptoms that may confound studies aiming to assess the association between insomnia and CVD risk.

## Supplementary Material

zsac318_suppl_Supplementary_Files
