# Supplementary material for: Association between insomnia phenotypes and subclinical myocardial injury: the Multi-Ethnic Study of Atherosclerosis
Source: Sleep. 2022 Dec 29;46(4):zsac318. doi: 10.1093/sleep/zsac318 (PMC10091090; doi:10.1093/sleep/zsac318)
Supplement: zsac318_suppl_Supplementary_Files [file zsac318_suppl_supplementary_files.docx]

**Association between insomnia phenotypes and subclinical myocardial injury:**

**The Multi-Ethnic Study of Atherosclerosis**

Fjola D. Sigurdardottir ^1,2^

Suzanne M. Bertisch ^3,4^

Michelle L. Reid ^3^

Christopher R. deFilippi ^5^

Joao A.C. Lima ^6^

Susan Redline ^3,4^

Torbjørn Omland ^1,2^

1. Department of Cardiology, Akershus University Hospital, Lørenskog, Norway.

2. Institute of Clinical Medicine, University of Oslo, Oslo, Norway.

3. Division of Sleep and Circadian Disorders, Brigham and Women's Hospital, Boston, Massachusetts.

4. Division of Sleep Medicine, Harvard Medical School, Boston, MA, USA.

5. Inova Heart and Vascular Institute, Inova Fairfax Medical Campus, Falls Church, VA.

6. Division of Cardiology, Department of Medicine, Johns Hopkins Hospital and School of Medicine, Baltimore, Maryland, USA.

Corresponding author:

Torbjørn Omland MD, PhD, MPH

Professor of Medicine, University of Oslo

Division of Medicine, Akershus University Hospital

NO-1478 Lørenskog, Norway

e-mail: torbjorn.omland@medisin.uio.no

**SUPPLEMENTARY TABLES**

**Supplementary table 1**. Association between insomnia symptoms in females and circulating cTnT in different insomnia phenotypes; insomnia symptoms, insomnia symptoms with actigraphy-defined short sleep duration (<6h), insomnia with fragmented sleep and COMISA with no symptoms of insomnia as a reference.

|  | **Model 1** | **Model 2** | **Model 3** | **Model 4** |
| --- | --- | --- | --- | --- |
|  | ß (SE) | ß (SE) | ß (SE) | ß (SE) |
| **No insomnia** | **Ref** | **Ref** | **Ref** | **Ref** |
| **Insomnia** | 0.09 (0.03)** | 0.03 (0.03) | 0.02 (0.03) | 0.03 (0.03) |
| **Insomnia with short sleep duration (< 6h)** | 0.18 (0.05)** | 0.08 (0.04) | 0.04 (0.04) | 0.07 (0.04) |
| **Insomnia with fragmented sleep (<20%)** | 0.12 (0.04)** | 0.05 (0.03) | 0.03 (0.03) | 0.05 (0.04) |
| **OSA (Defined as AHI > 15)** | 0.14 (0.03)** | 0.08 (0.03)** | 0.01 (0.03) | ----- |
| **COMISA** | 0.14 (0.04)** | 0.08 (0.04)* | 0.03 (0.04) | ----- |
| Model 1: Unadjusted Model 2: Age, race/ethnicity, smoking  Model 3: Model 2 + BMI  Model 4: Model 3 + eGFR, AHI (except for COMISA and OSA)  **p<0.01, *p<0.05 | | | | |

**Supplementary table 2**. Association between insomnia symptoms in males and circulating cTnT in different insomnia phenotypes; insomnia symptoms, insomnia symptoms with actigraphy-defined short sleep duration (<6h), insomnia with fragmented sleep and COMISA with no symptoms of insomnia as a reference.

|  | **Model 1** | **Model 2** | **Model 3** | **Model 4** |
| --- | --- | --- | --- | --- |
| **No insomnia** | **Ref** | **Ref** | **Ref** | **Ref** |
| **Insomnia** | 0.13 (0.03)** | 0.03 (0.03) | 0.02 (0.03) | 0.01 (0.03) |
| **Insomnia with short sleep duration (< 6h)** | 0.19 (0.05)** | 0.09 (0.04)* | 0.06 (0.04) | 0.06 (0.04) |
| **Insomnia with fragmented sleep (<20%)** | 0.12 (0.04)** | 0.03 (0.03) | 0.02 (0.03) | 0.03 (0.03) |
| **OSA (Defined as AHI > 15)** | 0.15 (0.04)** | 0.14 (0.03)** | 0.09 (0.03)** | ------ |
| **COMISA** | 0.16 (0.04)** | 0.09 (0.04)* | 0.06 (0.04) | ------ |
| Model 1: Unadjusted Model 2: Age, race/ethnicity, smoking  Model 3: Model 2 + BMI  Model 4: Model 3 + eGFR, AHI (except for COMISA and OSA) **p<0.01, *p<0.05 | | | | |

**Supplementary table 3:** Association between insomnia symptoms and circulating cTnT in different insomnia phenotypes; insomnia symptoms, insomnia symptoms with polysomnography evaluated short sleep duration (<6h), insomnia with an elevated arousal index (>28.1), and insomnia with increased polysomnography-defined wake after sleep onset (>94 minutes), with no symptoms of insomnia as a reference. **p<0.01, *p<0.05

|  | **Model 1** | **Model 2** | **Model 3** | **Model 4** |
| --- | --- | --- | --- | --- |
|  | ß (SE) | ß (SE) | ß (SE) | ß (SE) |
| **No insomnia with PSG sleep duration≥ 6h (N=591)** | **Ref** | **Ref** | **Ref** | **Ref** |
| **No insomnia with PSG short sleep duration (< 6h) (N=551)** | 0.11 (0.03)** | 0.02 (0.03) | 0.005 (0.03) | 0.01 (0.03) |
| **Insomnia with PSG sleep duration (≥ 6h) (N=501)** | 0.10 (0.03)** | 0.04 (0.03) | 0.03 (0.03) | 0.03 (0.03) |
| **Insomnia with PSG short sleep duration (< 6h) (N=545)** | 0.24 (0.03)** | 0.03 (0.03) | 0.004 (0.03) | 0.02 (0.03) |
| Model 1: Unadjusted  Model 2: Sex, age, race/ethnicity, smoking  Model 3: Model 2 + BMI  Model 4: Model 3 + eGFR, AHI  **p<0.01, *p<0.05 | | | | |

| **No insomnia with normal arousal index (N=1014)** | **Ref** | **Ref** | **Ref** | **Ref** |
| --- | --- | --- | --- | --- |
| **No insomnia with elevated arousal index (>28.1 ) (N =234)** | 0.13 (0.04)** | 0.04 (0.03) | 0.01 (0.03) | -0.04 (0.03) |
| **Insomnia with normal arousal index (N=679)** | 0.10 (0.03)** | 0.04 (0.02) | 0.03 (0.02) | 0.04 (0.02) |
| **Insomnia with elevated arousal index (>28.1) (N=261)** | 0.16 (0.04)** | 0.003 (0.03) | -0.03 (0.03) | -0.06 (0.03) |
| Model 1: Unadjusted  Model 2: Sex, age, race/ethnicity, smoking  Model 3: Model 2 + BMI  Model 4: Model 3 + eGFR, AHI  **p<0.01, *p<0.05 | | | | |

| **No insomnia with wake after sleep onset (PSG) < 94 (N=764)** | **Ref** | **Ref** | **Ref** | **Ref** |
| --- | --- | --- | --- | --- |
| **No insomnia with wake after sleep onset (PSG) >=94 (N=378)** | 0.19 (0.04)** | 0.03 (0.03) | 0.02 (0.03) | 0.02 (0.03) |
| **Insomnia with wake after sleep onset (PSG) < 94 (N=610)** | 0.11 (0.03)** | 0.03 (0.03) | 0.01 (0.02) | 0.02 (0.03) |
| **Insomnia with wake after sleep onset (PSG) >= 94 (N=436)** | 0.29 (0.03)** | 0.05 (0.03) | 0.03 (0.03) | 0.03 (0.03) |
| Model 1: Unadjusted  Model 2: Sex, age, race/ethnicity, smoking  Model 3: Model 2 + BMI  Model 4: Model 3 + eGFR, AHI  **p<0.01, *p<0.05 | | | | |
